# Towards facilitated interpretation of shotgun metagenomics long-read sequencing data analyzed with KMA for the detection of bacterial pathogens and their antimicrobial resistance genes

**DOI:** 10.3389/fmicb.2024.1336532

**Published:** 2024-04-04

**Authors:** Mathieu Gand, Indre Navickaite, Lee-Julia Bartsch, Josephine Grützke, Søren Overballe-Petersen, Astrid Rasmussen, Saria Otani, Valeria Michelacci, Bosco Rodríguez Matamoros, Bruno González-Zorn, Michael S. M. Brouwer, Lisa Di Marcantonio, Bram Bloemen, Kevin Vanneste, Nancy H. C. J. Roosens, Manal AbuOun, Sigrid C. J. De Keersmaecker

**Affiliations:** ^1^Transversal Activities in Applied Genomics, Sciensano, Brussels, Belgium; ^2^Department of Bacteriology, Animal and Plant Health Agency, Weybridge, United Kingdom; ^3^Department of Biological Safety, German Federal Institute for Risk Assessment, Berlin, Germany; ^4^Bacterial Reference Center, Statens Serum Institute, Copenhagen, Denmark; ^5^National Food Institute, Technical University of Denmark, Kongens Lyngby, Denmark; ^6^Department of Food Safety, Nutrition and Veterinary Public Health, Istituto Superiore di Sanità, Rome, Italy; ^7^Department of Animal Health, Complutense University of Madrid, Madrid, Spain; ^8^Wageningen Bioveterinary Research Part of Wageningen University and Research, Lelystad, Netherlands; ^9^Istituto Zooprofilattico Sperimentale dell’Abruzzo e del Molise “G. Caporale”, Teramo, Italy

**Keywords:** metagenomics, ONT, bioinformatics, pathogens, antimicrobial resistance, KMA, database, results interpretation

## Abstract

Metagenomic sequencing is a promising method that has the potential to revolutionize the world of pathogen detection and antimicrobial resistance (AMR) surveillance in food-producing environments. However, the analysis of the huge amount of data obtained requires performant bioinformatics tools and databases, with intuitive and straightforward interpretation. In this study, based on long-read metagenomics data of chicken fecal samples with a spike-in mock community, we proposed confidence levels for taxonomic identification and AMR gene detection, with interpretation guidelines, to help with the analysis of the output data generated by KMA, a popular *k-*mer read alignment tool. Additionally, we demonstrated that the completeness and diversity of the genomes present in the reference databases are key parameters for accurate and easy interpretation of the sequencing data. Finally, we explored whether KMA, in a two-step procedure, can be used to link the detected AMR genes to their bacterial host chromosome, both detected within the same long-reads. The confidence levels were successfully tested on 28 metagenomics datasets which were obtained with sequencing of real and spiked samples from fecal (chicken, pig, and buffalo) or food (minced beef and food enzyme products) origin. The methodology proposed in this study will facilitate the analysis of metagenomics sequencing datasets for KMA users. Ultimately, this will contribute to improvements in the rapid diagnosis and surveillance of pathogens and AMR genes in food-producing environments, as prioritized by the EU.

## 1 Introduction

In the context of diagnosis, the methods currently used for pathogen detection and characterization and outbreak investigation or surveillance are mainly reliant on culture-based approaches that are time-consuming. These conventional techniques are targeted and hence limited to few pathogens and their related antimicrobial resistance (AMR) and virulence genes, all prioritized based on their level of risk or pathogenicity. Additionally, only one or a few isolates obtained from the sample are often characterized by culture-based methods. Thus, these approaches cannot provide the full picture of all the pathogens and AMR genes present in a sample. The increasing burden of AMR is not limited to healthcare systems but spreads also in food-producing environments. Considering this, untargeted techniques are needed for surveillance and rapid detection of resistant food-borne pathogens from a One Health perspective (Torres et al., 2021; Pennone et al., 2022; Pillay et al., 2022). Shotgun metagenomics, i.e., untargeted sequencing of all DNA material present in a sample, is a valuable tool to profile the composition of microbial communities for the identification of potential pathogens and AMR genes, as shown with short read-sequencing (Illumina) (Bengtsson-Palme, 2017; Oniciuc et al., 2018; Pillay et al., 2022). Therefore, the EFSA BIOHAZ panel considered, in one of their opinions, the use of this technology for outbreak investigation and risk assessment (EFSA Panel on Biological Hazards et al., 2019). Moreover, with this technique, the selection and dissemination of AMR in microbiomes of food-producing animals and their environments can be better studied using, for instance, fecal samples as a proxy, to track horizontal gene transfer between commensal and pathogenic bacteria (Bengtsson-Palme, 2017; Torres et al., 2021). Long-read sequencing (Oxford Nanopore Technologies, ONT) can also be used for pathogen and AMR detection by shotgun metagenomics (Pennone et al., 2022; Pillay et al., 2022). Oxford Nanopore technology is portable, generating sequencing results in real-time and making on-site use possible, without the need to send samples to centralized sequencing laboratories using big laboratory equipment (Leggett and Clark, 2017). This has the potential to facilitate real-time detection of pathogens carrying AMR genes in the food chain, to implement appropriate treatment options and improve risk assessment by monitoring the spread of AMR genes within the microbiome (Gardy and Loman, 2018; Lamb et al., 2020).

Due to the huge amount of data generated by shotgun metagenomics sequencing, it is essential to have efficient bioinformatics tools for data analysis. Moreover, the relatively easy use of the ONT technology needs to be combined with easy and unbiased data interpretation. Several bioinformatics tools and databases exist for bacterial taxonomic identification and AMR gene detection (Breitwieser et al., 2019; Pillay et al., 2022). They can roughly be separated into two groups: read-mapping-based and assembly-based. The assembly-based approach can be resource-demanding, and in the context of gene detection, poor assembly quality or gene split between multiple contigs can return erroneous results (Pillay et al., 2022). The direct mapping of reads against large databases containing reference genomes or genes of interest has been demonstrated to be accurate, fast, and resource-efficient when applied to Illumina sequencing data from bacterial isolates (Clausen et al., 2016). The open-source bioinformatics tool *k*-mer alignment (KMA) is a fast read mapper using *k*-mer alignment and has been proven to be more performant in accuracy and speed in comparison with other classifiers (Clausen et al., 2018; Marcelino et al., 2020). Notably, it has been widely used in studies for metataxonomics (16S/18S rRNA) and AMR gene identification from metagenomics samples sequenced with short-read technologies (Sturød et al., 2020; Horie et al., 2021; Speksnijder et al., 2022; Stege et al., 2022). Using a scoring scheme named ConClave, KMA is able to overcome the difficulties encountered when using a redundant database, such as SILVA for metataxonomics and ResFinder for AMR detection (Clausen et al., 2018). Recently, KMA has also been integrated into the CCMetagen pipeline, to improve output data filtering and visualization, and applied to taxonomic classification using large databases containing full genomes (Marcelino et al., 2020). Finally, KMA has also been employed with long-read technology in metagenomics studies including targeted sequencing, DNA enrichment, and in combination with short-reads (Kinoshita et al., 2021; Kirstahler et al., 2021; D’aes et al., 2022; Schulz et al., 2022; Teudt et al., 2022). Based on these observations, KMA is an efficient tool to be included in a rapid protocol for bacterial species and AMR gene identification based on sequencing approaches. However, in the aforementioned studies using KMA, a large variety of settings and confidence thresholds were used to analyze and interpret the data, often without a clear justification of what motivated the choice of these criteria. Indeed, the choice of optimal parameters and database, to use with bioinformatics tools and the validated confidence thresholds to filter the output data and avoid false positive results, is not always clearly communicated or extensively tested (Breitwieser et al., 2019; Wright et al., 2023). However, these parameters have a critical impact on the results and their correct interpretation. Moreover, the interpretation of the bioinformatics output results, usually aiming at answering as simple questions as “Does my sample contain pathogen(s) carrying AMR gene(s)?,” is not always straightforward. Therefore, more knowledge is needed about the impact of parameters, thresholds, and databases on the generation of true and false positive results when using bioinformatics tools, such as KMA (Smith et al., 2022; Wright et al., 2023; Xu et al., 2023). Furthermore, guidelines for clear and easy interpretation, which are currently lacking, are needed to allow reliable detection of pathogens and their AMR genes in metagenomics samples. Finally, KMA has mostly been used with short-read sequence data, and more studies are needed to investigate the feasibility of using this tool with long reads solely for pathogen and AMR detection by shotgun metagenomics.

In the present study, we aimed to facilitate for KMA users the interpretation of the analysis results that are generated from ONT metagenomics for bacterial taxonomic identification and AMR detection. To this end, we first evaluated the impact of the completeness of the template genomes in the KMA database on the correct interpretation of the species identification, leading to some guidelines on which the database is used. Subsequently, we proposed confidence thresholds to filter the KMA output data for the correct detection of pathogens potentially carrying AMR genes, with recommendation for complementary analyses to confirm or reject the detection if needed. To make this evaluation, we used defined microbial mock communities of known composition, abundance and AMR gene content. These mock communities were used in “pure” status and “spiked” in chicken fecal material. The obtained samples were sequenced using ONT (long-read) with different levels of multiplexing, and analyzed with KMA for taxonomic classification and AMR gene detection. The “unspiked” matrix was also analyzed. From these experiments, KMA settings and confidence threshold were selected and subsequently tested on 28 sequencing datasets. Additionally, we explored the possibility of using KMA to link detected AMR genes to their bacterial host chromosome.

## 2 Materials and methods

### 2.1 Sample preparation

A commercial defined microbial mock community, containing 14 bacteria, one archaeon, and their related AMR genes, if any, at various concentrations (Supplementary material S1), was used throughout this study: the ZymoBIOMICS Gut Microbiome Standard D6331 (GMS) (Zymo Research, Irvine, United States). Five different strains of *Escherichia coli* were present in the standard, each at 2.8%, reaching an abundance of 14% for the species. Although the GMS also contains two fungal strains (not shown in Supplementary material S1), those fungal strains were not investigated as the present study focused on bacteria and detection of their AMR genes. Species composition of the GMS and relative abundance were provided by the manufacturer. AMR gene composition of each GMS strain was determined by analyzing their reference genome sequence (as communicated by the manufacturer: https://s3.amazonaws.com/zymo-files/BioPool/D6331.refseq.zip; accessed in January 2022) and performing AMR gene detection (at the nucleotide level), as described by Bogaerts et al. (2019) against the ResFinder database (Zankari et al., 2012) with the following parameters: minimum coverage 60% and minimum percentage identity 90%. All identified AMR genes were found with 100% coverage and more than 97% of identity (Supplementary material S2).

The GMS was either analyzed “pure” or “spiked” into a matrix. For the “pure” condition, 75 μL of the GMS (≈ 2.96 × 10^8^ cells in total, stored in DNA/RNA shield 2x concentrate) was mixed with 75 μL of DNA/RNA nuclease-free distilled water (Thermo Fisher Scientific, Waltham, United States) and 50 μL of DNA/RNA shield 1x (R1100; Zymo Research, Irvine, United States). The same was applied for the “spiked” condition, except for that 100 mg of chicken fecal material was added to the mixture. Finally, a third mix, named “unspiked,” was prepared with 100 mg of chicken fecal material (from the same fecal sample used for “spiked” condition) and 200 μL of DNA/RNA shield 1x (R1100; Zymo Research, Irvine, United States).

### 2.2 DNA extraction

Total genomic DNA was extracted from the “pure,” “spiked,” and “unspiked” samples using a kit previously used for metagenomics ONT sequencing [Quick-DNA HMW MagBead kit (Zymo Research, Irvine, United States; Gand et al., 2023)] with the following modifications: (i) during microbial lysis, enzymatic digestion (step 6) was performed with 20 μL of a 5 mg/mL solution of MetaPolyzyme (Sigma–Aldrich, Saint-Louis, United States) for 1 h at 37°C (instead of lysozyme), and proteinase K digestion (step 9) was performed for 30 min; (ii) during DNA purification, incubation time with the MagBinding Beads (step 4) was extended to 20 min, and elution (step 18) was performed for 10 min at 55°C to maximize the recovery of long fragments.

The DNA yield and fragment length were measured using the Invitrogen Qubit 4 Fluorometer (Thermo Fisher Scientific, Waltham, United States) with Qubit dsDNA HS Assay Kit (Thermo Fisher Scientific, Waltham, United States) and the 4200 TapeStation System (Agilent, Santa Clara, United States), with the Genomic DNA ScreenTape and reagents (Agilent, Santa Clara, United States), respectively. For “spiked” and “unspiked” samples, sufficient DNA, with average fragment size comprised between 56,000 bp (“unspiked” sample) and > 60,000 bp (“spiked” sample), was obtained for the preparation of two ONT libraries. Concerning the “pure” GMS sample, DNA extraction was performed twice to get enough DNA for the two libraries, and the obtained average fragment length was comprised between 29,000 bp (1^st^ extract) and 37,000 bp (2^nd^ extract) (Supplementary material S3).

### 2.3 ONT sequencing

For the singleplex experiment, the library preparation was performed using the Ligation sequencing kit for genomic DNA (SQK-LSK109) (Oxford Nanopore Technologies, Oxford, United Kingdom) with 1 μg of DNA as input. The generated libraries were then loaded on a Spot-ON MinION flow cell (FLO-MIN 106D, R9.4.1 version) (Oxford Nanopore Technologies, Oxford, United Kingdom) with one sample (“pure,” “spiked,” or “unspiked”) per flow cell. Sequencing was performed on the Mk1C device (Oxford Nanopore Technologies, Oxford, United Kingdom) for 72 h with live-basecalling turned off. The same method was used for the triplex experiment, except that the native barcoding expansion kit (EXP-NBD104) (Oxford Nanopore Technologies, Oxford, United Kingdom) was used for multiplexing the “pure,” “spiked,” and “unspiked” samples into a barcoded library which was loaded onto one MinION flow cell.

Raw sequencing data were basecalled and demultiplexed using Guppy version 5.0.7 (Oxford Nanopore Technologies, Oxford, United Kingdom) on a GPU server in super high accuracy mode with config file dna_r9.4.1_450bps_sup.cfg. Trim strategy was set to DNA, and qscore filtering was disabled. The basecalled reads were subsequently filtered with NanoFilt version 2.8.0 (De Coster et al., 2018), to obtain high-quality reads with minimum quality score and length equal to 10 and 500 bp, respectively. The data are available on the NCBI Sequence Read Archive (SRA) repository under the BioProject ID PRJNA1031997. Statistics of the filtered high-quality reads were obtained with NanoPlot version 1.36.2 (De Coster et al., 2018) (Supplementary material S4).

### 2.4 KMA databases

*k*-mer alignment requires a reference database that is indexed. This database contains the template reference sequences against which the reads will be mapped. Some of the KMA databases used in this study (abbreviated with the nomenclature “name_db” throughout the manuscript) were downloaded, already indexed, and ready to use. Alternatively, sequences (FASTA format) were indexed into a KMA database using kma_index (included in KMA tool; Clausen et al., 2018) with default options. All the databases used in this study are available upon request.

The GMS_db is composed of the genome sequences belonging to the different strains included in the GMS. This database was indexed using the sequences provided by ZymoResearch and was accessible here: https://s3.amazonaws.com/zymo-files/BioPool/D6331.refseq.zip (accessed in January 2022).

The RefSeqFull_db and RefSeqComplete_db were indexed with kma_index from sequences downloaded in January 2019 from NCBI and manually curated at Danmarks Tekniske Universitet (DTU) using the criteria described by Osakunor et al. (2020). In brief, the sequences were downloaded via NCBI GenBank clade specific assembly_summary.txt files.^1^ The sequences were selected from the assembly_summary.txt file with the tags version_status = “latest,” genome_rep = “Full,” and assembly_level = “Complete genome” or “Chromosome.” Then, the additional tag refseq_category = “representative genome” for the RefSeqFull_db was also selected. As such, the RefSeqComplete_db was composed of complete bacterial genomes, i.e., complete and chromosome assembly levels only, and the RefSeqFull_db was composed of complete and partial bacterial genomes, i.e., complete, chromosome, scaffold, and contig assembly levels. No plasmids were included. Next, almost identical genomes were removed from the two databases by filtering out with dRep (Olm et al., 2017); all sequences had more than 99% of similarity with other sequences, keeping only one each time. Finally, the RefSeqComplete_db was trimmed to keep only clinically relevant species, i.e., all the strains from the Two Weeks in the World (TWIW) collection (Nag et al., 2023). After curation, the RefSeqFull_db was composed of 944,390 sequences belonging to 18,999 unique species that are shown in Supplementary material S5. The RefSeqComplete_db was composed of 12,335 sequences belonging to 2,926 unique species that are shown in Supplementary material S6.

The RefSeqComplete_db did not contain sequences belonging to three of the GMS species, i.e., *Veillonella rogosae*, *Prevotella corporis* (no complete genome sequences available in NCBI at the time of the database construction), and *Methanobrevibacter smithii* (filtered out during the trimming of the database, see above). The RefSeqFull_db did not contain sequences belonging to one of the GMS species, i.e., *Veillonella rogosae*. The farmed_db was composed of the RefSeqComplete_db, to which RefSeq complete genome sequences belonging to *M. smithii* (GCF_000016525.1, GCF_002813085.1, GCF_022846175.1, and GCF_022846155.1) and RefSeq draft incomplete genome sequences (contigs or scaffolds) belonging to *V. rogosae* (GCF_002959775.1) and *P. corporis* (GCF_001546595.1, GCF_000430525.1, and GCF_022713225.1), three GMS species which were missing from this database, were appended using the option -t_db in kma_index. For *V. rogosae* and *P. corporis*, no complete genome sequences were available in RefSeq when the farmed_db was created (June 2022).

Finally, for antimicrobial resistance, the ResFinder database (ResFinder_db of August 2020) was used to assign antimicrobial resistance genes to all our microbiomes.

### 2.5 KMA analysis

*k*-mer alignment version 1.4.4 was used with the filtered long-read sequencing data, and the following options are described hereinafter. Circular alignment was allowed with -ca. Each read was mapped to only one template with −1 t1 (preferred for the determination of a “best hit,” see below). The -proxi parameter was set to 0.9. Additionally, the minimum template identity (-ID) was set loose to 0.01, as the output data were subsequently evaluated according to confidence threshold (see section 3.2). The option -mem_mode was included to save memory because large databases were used. In addition, to save computational time and resources, no alignment (−na) and consensus (−nc) files were generated. Finally, KMA settings optimized for long reads included -bc set to 0.7 and -bcNano (https://bitbucket.org/genomicepidemiology/kma/src/master/README.md; accessed on June 2023). The detailed specification of KMA can be assessed here: https://bitbucket.org/genomicepidemiology/kma/src/master/KMAspecification.pdf; accessed on June 2023. From the result file (.res) generated by KMA (available upon request), the “best hit” per bacterial species or AMR gene, i.e., the template having the highest depth, was extracted with its corresponding mapping statistics: template name, template identity, template length, and depth value. The result (.res) file containing these statistics can be imported and further processed in any tabular software (such as Excel), where the confidence thresholds developed in section 2.6 can be applied to filter the KMA template results per level of confidence detection.

### 2.6 Determination of KMA confidence threshold using specificity evaluation

The detection confidence thresholds were determined using the true positive (TP) and false positive (FP) hits. For the “pure” and “spiked” samples, when KMA reported mapping statistics (depth, template identity, and template length) for a best hit belonging to one of the species or AMR included in the GMS, this was considered as a TP. For the “pure” sample, when KMA reported mapping statistics for a best hit not belonging to one of the GMS species or their AMR, this was considered as an FP. The investigation of FP was not performed for the “spiked” sample as the background microflora contained non-GMS species, which were unknown. Based on this result clustering excluding the FP while including the TP, the confidence thresholds were derived from the mapping statistics.

### 2.7 Linking AMR gene detection and species identification

To establish the link between AMR gene detection and species identification, a two-step KMA procedure was performed with the steps presented in this section. The first-step KMA analysis was performed using the command line described in section 2.5 and a taxonomic database, i.e., the GMS_db or the farmed_db, for bacterial species detection. Then, all the reads attributed to the species of interest were extracted from the sequencing dataset. For doing this, all the read identifiers linked to this species were extracted from the fragment file (.frag) and generated by KMA (using grep with “species name” as argument and cut to extract the read ID from the 7^th^ field of each line retrieved by grep), to make an identifier list. Then, this list of identifiers was subsequently used as input in seqtk_subset (version 1.2; https://github.com/lh3/seqtkhttps://github.com/lh3/seqtk), to extract the species reads from the original dataset, which was used as input in the first KMA analysis. The extracted reads formed a new dataset that was specific for the considered species and were finally used as input in the second-step KMA analysis by the same command line as described in Section 2.5, but this time with the ResFinder_db for AMR gene identification. The second-step KMA analysis was repeated for each species of interest for which the reads were extracted from the original sequencing dataset as described above.

With the aim to estimate the time that the analysis would need to be completed locally (without Internet required) on a laptop, if used with an on-site portable setting, the two-step KMA procedure was tested on a virtual machine equipped with eight virtual CPUs, 8 GB of RAM, and 150 GB of disk space.

### 2.8 Evaluation and validation of the applicability of the KMA confidence thresholds with various metagenomics datasets

The confidence thresholds selected in Section 3.2 for taxonomic identification and AMR detection, as well as the two-step KMA method for species-AMR linkage (Section 2.7), were tested on different datasets. Some datasets including the sequencing of the GMS were publicly available or generated in the context of this study. Other publicly available datasets included sequencing data from two other mock communities: the Microbial Community Standard I (MCSI) and Microbial Community Standard II (MCSII) from ZymoResearch (D6300 and D6310, respectively). This Microbial Community Standard was composed of eight bacterial strains in even (MCSI) or log (MCSII) distribution (Nicholls et al., 2019; Gand et al., 2023), and its AMR composition was obtained with ResFinder as described in section 2.1 for the GMS (Supplementary material S7).

Various experimental conditions, numbered from A to K in Table 1, were used for the generation of 21 out of the 28 datasets. Experiments B and C were used in Section 3.2 for the selection of the KMA threshold with the GMS. Experiments A, D, and E, which were obtained within the context of the present study, used the GMS “pure” or “spiked” in a matrix (chicken feces, buffalo feces, and pig feces), but with different experimental settings (Table 1), which are presented in more details in Supplementary material S8. Experiment A included the same samples but sequenced with Illumina technology. For each of these experiments, the matrix was also analyzed as “unspiked.” Therefore, Experiments A to E included three datasets, namely, “pure,” “spiked,” and “unspiked.” Experiments F and G were composed of publicly available datasets generated from the sequencing of the GMS (pure) with two different library preparation kits: kit9 (SQK-LSK109) vs. kit12 Q20+ (SQK-LSK112) (Liu et al., 2022). Finally, experiments H to K were publicly available datasets encompassing the sequencing of the MCSI and MCSII, which analyzed pure or spiked in a synthetic fecal matrix, with various experimental settings (Nicholls et al., 2019; Gand et al., 2023; Table 1).

**Table 1:** Summary of the experimental settings used for the generation of the different sequencing datasets including the GMS, MCSI, and MCSII.

											
Exp	Mock com.	Matrix	DNA extraction kit					Seq.		Library preparation kit				Multiplexing				Run. time			Basecalling			Q filt.				Acc. number	Ref.
			HMW	Zymo	Beck	Qia	CB	ONT	Illum	Ligat9	Ligat12	Rap	Next	1	3	5	6	24	48	72	fast	hac	sup	7	9	10	20		
A	GMS	Chicken feces*	●						●				●	●													●	PRJNA1031997	This study
B	GMS	Chicken feces*	●					●		●				●						●			●			●		PRJNA1031997	This study
C	GMS	Chicken feces*	●					●		●					●					●			●			●		PRJNA1031997	This study
D	GMS	Buffalo feces*	●					●		●						●			●		●			●				PRJNA1031997	This study
E	GMS	Pig feces*			●			●				●					●	●				●			●			PRJNA1031997	This study
F	GMS	-				●		●		●				●				†					●			●		SRR17907852	Liu et al. (2022)
G	GMS	-				●		●			●			●				†					●			●		SRR17907851	Liu et al. (2022)
H	MCSI even	-		●				●		●				●					●		‡			¥				ERR 3152364	Nicholls et al. (2019)
I	MCSII log	-		●				●		●				●					●		‡			¥				ERR 3152366	Nicholls et al. (2019)
J	MCSII log	Synthetic feces	●					●		●				●						●				●				SRR24138602	Gand et al. (2023)
K	MCSII log	Synthetic feces					●	●		●				●						●				●				SRR24138605	Gand et al. (2023)

Exp: experiments identifiers numbered from A to K; Mock com: Mock community used in the different experiments; Matrix: Matrix in which the mock community was spiked, when “*” was indicated, the GMS was tested “pure” or “spiked” into a matrix and the matrix was also tested “unspiked,” when “-“is indicated, the corresponding mock community was used “pure” only; DNA extract. Kit: DNA extraction kit with “HMW” being Quick-DNA HMW MagBead kit (Zymo Research), “Zymo” being ZymoBIOMICS DNA Miniprep extraction kit (Zymo Research), “Beck” being GenFindV3 (Beckman Coulter), “Qia” being Dneasy PowerSoil Kit (Qiagen) and “CB” being DNAexpress kit (Claremont BioSolutions); Seq: Sequencing platform with “ONT” being Oxford Nanopore Technology and “Illum” being Illumina; Library preparation kit: “Ligat9” and “Ligat12” being Ligation sequencing kit for genomic DNA SQK-LSK109 and SQK-LSK112 (Oxford Nanopore Technologies), respectively, “Rap” being Rapid Barcoding Kit (SQK-RBK004; Oxford Nanopore Technologies) and “Next” being Nextera XT kit (Illumina); Multiplexing: number of samples multiplexed during a sequencing run; Run time: sequencing running time in hours, only for ONT experiments (†: not communicated) as for Illumina this corresponds to the number of cycles, i.e., 2 × 250 bp run, and it is not comparable with long-read sequencing; Basecalling: basecalling model used in Guppy with “hac” being high accuracy and “sup” being super high accuracy (‡: former flipflop model was used); Q filt.: filtering of the basecalled data based on minimum quality (Q) score for Q7, Q9, and Q10, and on average quality score for Q20 (¥: Q score filtering was not communicated).

Furthermore, seven publicly available ONT metagenomics datasets out of the 28 datasets used in this study were also used for the validation of the KMA confidence thresholds and AMR linkage. Four out of these seven datasets were generated in the context of the detection of genetically modified microorganism (GMM), i.e., *Bacillus* species, *B. licheniformis*, *B. amyloliquefaciens,* and *B. velezensis*, in four food enzyme products (FE) (D’aes et al., 2022). Three out of the seven publicly available ONT datasets were produced with MinION and Flongle, to simulate a foodborne outbreak investigation for the detection of Shiga toxin-producing *Escherichia coli* (STEC) spiked in minced beef (MB) (Buytaers et al., 2021). The methodology developed in this study for KMA output data interpretation was applied to these seven datasets, and the generated data were compared with the expected results obtained in the original studies by D’aes et al. (2022) and Buytaers et al. (2021).

Sequencing statistics of the different experiments are presented in Supplementary material S4, and detailed protocol associated with the publicly available data presented in this section is accessible in the corresponding original studies (Table 1) (Nicholls et al., 2019; Buytaers et al., 2021; D’aes et al., 2022; Liu et al., 2022; Gand et al., 2023).

## 3 Results

### 3.1 Influence of the completeness of the template genomes in the KMA database on the correct result interpretation for detection of species

The composition of databases used in taxonomic classifier tools can greatly influence the output results and how they are reported and interpreted by users. Here, the impact of using the RefSeqComplete_db composed of complete genomes, i.e., one template sequence per bacterial genome in the database, or the RefSeqFull_db composed of a mixture of complete and draft genomes, i.e., incomplete assemblies resulting into a list of contigs or scaffolds serving as multiple templates for a given bacterial genome, was tested. The sequencing data of the defined microbial mock community GMS “pure” sample obtained with the ONT singleplex experiment (Exp B, Table 2) were analyzed using KMA with the two databases and the GMS_db for reference comparison. The mapping results of three GMS species present at different abundance levels, i.e., *Bacteroides fragilis* (14%), *Fusobacterium nucleatum* (6%), and *Akkermansia muciniphila* (1.5%), were compared. In addition to their diverse spiking concentrations, these species were selected because both complete and draft genomes belonging to them were present in the RefSeqFull_db. For each of these species, the mapping results belonging to the best KMA hit, i.e., the template sequence having the highest depth, are shown in Table 2.

**Table 2:** Comparison of KMA output results influenced by database composition.

			Complete genomes					Draft genomes					
DB	GMS species	Relative abundance^1^	Number of hits^2^	Best hit template name^3^	Length (bp)	Depth	ID%	Number of hits^2^	Best hit template name^3^	Number of scaffold^4^	Length (bp)	Depth	ID%
**GMS_db**	*Bacteroides fragilis*	14	1/1	Bacteroides _fragilis	5.17 × 10^6^	503.15	99.97	-	-	-	-	-	-
	*Fusobacterium nucleatum*	6	1/1	Fusobacterium _nucleatum	2.45 × 10^6^	240.12	99.99	-	-	-	-	-	-
	*Akkermansia muciniphila*	1.5	1/1	Akkermansia_muciniphila	2.85 × 10^6^	129.27	99.99	-	-	-	-	-	-
**RefSeq** **Complete_db**	*Bacteroides fragilis*	14	7/7	CP012706.1	4.90 × 10^6^	355.39	94.01	-	-	-	-	-	-
	*Fusobacterium nucleatum*	6	7/16	NC_021281.1	2.26 × 10^6^	145.35	91.86	-	-	-	-	-	-
	*Akkermansia muciniphila*	1.5	4/6	CP024742.1	2.76 × 10^6^	116.79	97.99	-	-	-	-	-	-
**RefSeqFull_db**	*Bacteroides fragilis*	14	9/14	CP012706.1	4.90 × 10^6^	113.72	70.52	57/377	MGS47 scaffold84	36/118	7.39 × 10^4^	473.4	99.45
	*Fusobacterium nucleatum*	6	6/23	CM002368.1	2.15 × 10^6^	10.4	39.6	4/554	ATCC 51191 scaffold294	4/465	9.63 × 10^2^	11.24	100
	*Akkermansia muciniphila*	1.5	9/27	CP025827.1	2.76 × 10^6^	107.55	97.49	8/81	MGS154 scaffold65	8/81	7.43 × 10^4^	4.38	39.84

DB: Database; Length: Template length in bp; ID%: Template identity in percent; −: not applicable. ^1^Percentage of genomic DNA from each species in the GMS (according to the manufacturer). ^2^Number of templates reported by KMA out of the total number of sequences available in the database, for the corresponding species. ^3^NCBI accession numbers for RefSeqComplete_db and RefSeqFull_db and strain names for GMS_db. ^4^Number of templates reported by KMA out of the total number of scaffold sequences available in the database and belonging to the corresponding template strain name. The table shows the template length, template identity, and depth of the templates having obtained the maximum depth (best hit) among the results reported by KMA, from the complete draft genomes present in the tested databases, for the considered species.

As expected, high depth and high template identity (> 99.9%) were obtained when analyzing the “pure” sequencing data with the GMS_db. Moreover, the depth declined with respect to the decreasing relative abundance of the considered species. Comparable results (high depth and identity) were observed when the KMA analysis was performed with the RefSeqComplete_db. A global decrease in the depth and template identity was observed for complete genomes when the “pure” sample was analyzed with the RefSeqFull_db, but this was particularly dramatic for *B. fragilis* and *F. nucleatum*. For these two species, best hits from RefSeqFull_db corresponding to draft genomes obtained a higher depth and template identity than hits corresponding to complete genomes. However, it can be noted that the scaffold sequences from these draft genomes, which were determined as best hits, have a length that covers only 1.5% (MGS47 scaffold84) and 0.04% (ATCC 51191 scaffold294) of the corresponding complete genomes from the same species, i.e., CP117955.1 and CP117964.1, respectively. As shown in Supplementary material S9 for *B. fragilis*, templates with short length were also more likely to obtain high mapping scores when other GMS species were considered (data not shown). Moreover, when using the RefSeqFull_db, reads mapped to only a fraction of the total number of contigs belonging to one bacterial strain from the database, only 31 and 0.9% of the total available scaffolds belonging to *B. fragilis* MGS47 and *F. nucleatum* ATCC 51191, respectively, were reported with mapping scores (Table 2). Therefore, the interpretation of KMA mapping results should be done with caution when using draft genomes, preferably compiling all the results from template contigs belonging to the same bacterial genome (assembly), otherwise the species detection results can be misleading.

The decrease in depth and template identity observed for complete genome templates when using the RefSeqFull_db, in comparison with RefSeqComplete_db (Table 2), could be explained by different templates being reported as best hit between the two databases, potentially not having the same specificity for the strains present in the GMS. Nevertheless, this is not true for *B. fragilis* as the best hit template (CP012706.1) was identical for the two databases. Another hypothesis is that the reads have mapped to multiple templates, i.e., multiple scaffold sequences, instead of being grouped to one perfect match. This is supported by the data presented in Supplementary material S9 showing that the scaffold sequences have obtained a wide range of depth and template identity values, which, as a result, decreased mapping statistics for CP012706.1 when analyzed with RefSeqFull_db. On the contrary, when using the RefSeqComplete_db, only one out of the seven *B. fragilis* templates reported by KMA (Table 2; Supplementary material S9) obtained high mapping scores, suggesting that most of the reads mapped to CP012706.1 and allowing the clear determination of a best match. As the farmed_db is derived from the RefSeqComplete_db, to which missing GMS species were added, the same ease of interpretation was obtained for this database (Supplementary material S10). Therefore, the farmed_db was preferably used in sections 3.2, 3.4, and 3.5 for the elaboration of the confidence threshold and their application on various datasets.

### 3.2 Determination of confidence threshold for a clear and easy interpretation of KMA output data

**Figure 1:**
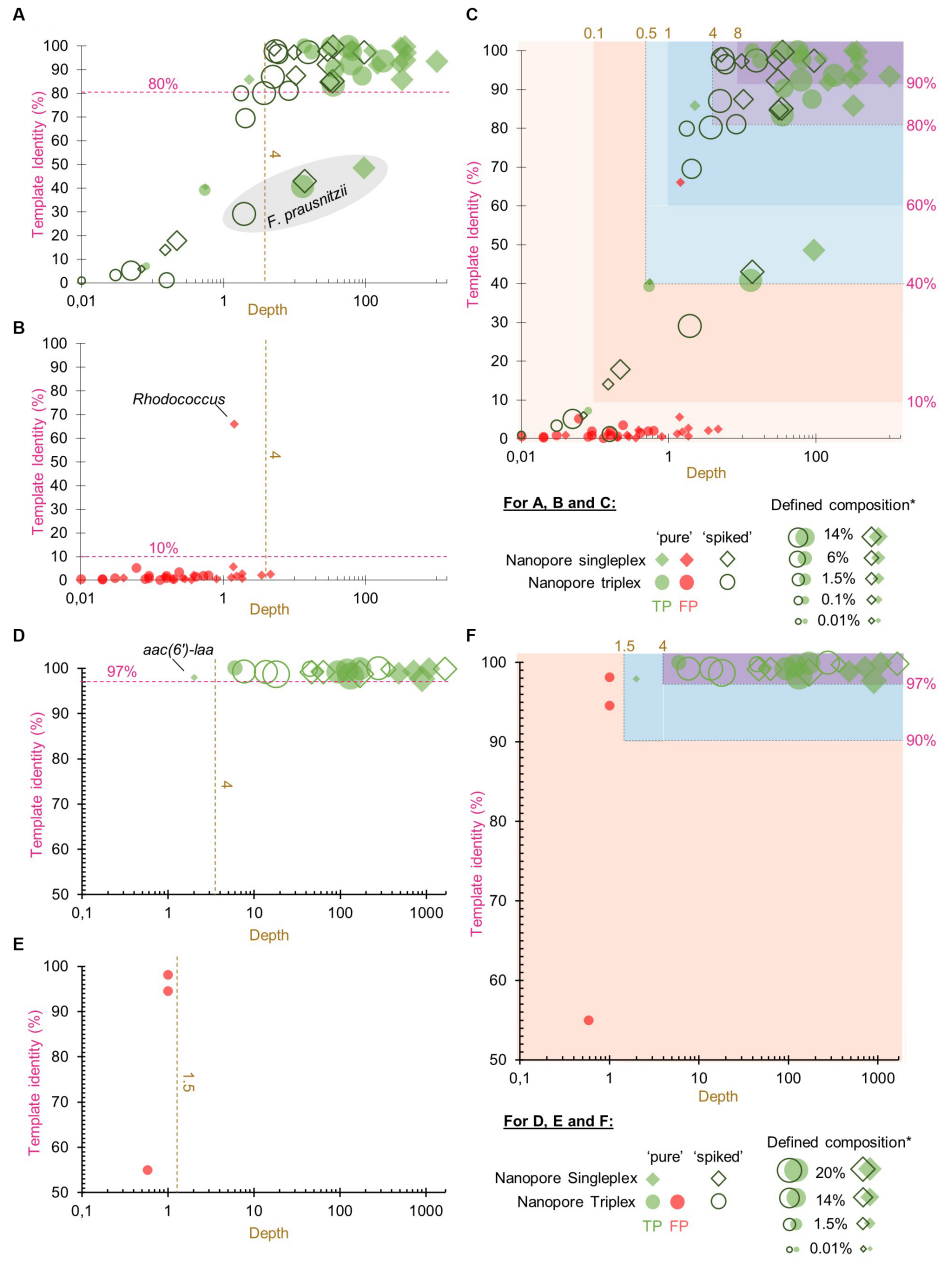
True positive and false positive results reported by *k*-mer alignment (KMA) for the detection of species and AMR to define confidence thresholds. The graphs show the representation of the template identity (*y*-axis) in function of the depth (*x*-axis in log scale) for all the TP (parts **A,D**) and FP (parts **B,E**) best hits reported by KMA when analyzing the sequencing data of the “pure” and “spiked” samples obtained by ONT singleplex and triplex. Parts **(A–C)** show the results obtained with the farmed_db and parts **(D–F)** show the results obtained with the ResFinder_db. The gray shape in part **(A)** shows the mapping results obtained for the species *Faecalibacterium prausnitzii*. Parts **(C,F)** show superposition of the TP and FP value and proposition of different levels of confidence threshold for the detection of species and AMR genes based on depth and template identity. ^*^defined composition represents the percentage of genomic DNA from each species in the GMS, as communicated by the manufacturer.

To help users to interpret the KMA output, confidence thresholds were derived from experimental data. “Pure” and “spiked” GMS samples, sequenced by ONT singleplex (Experiment B, Table 1) and triplex (Experiment C, Table 1), were analyzed by KMA using the farmed_db (for the detection of species) and ResFinder_db (for the detection of AMR genes). The best hits from the output data were classified into TP or FP results (Figure 1), according to the expected results from the GMS, i.e., composition of species and AMR genes (Supplementary material S1).

Among all the detected GMS species in the four conditions (52 in total), the majority of the TP best hits (34/52, 65%) were grouped at the top right of the graph, as shown in Figure 1A, with depth values above 4 and percentage identities above 80% (Supplementary material S11). This group was predominantly composed of the most abundant species: 75% of the five species spiked at 14% over the 4 conditions (15/20) and 81% of the four species spiked at 6% over the four conditions (13/16). Nevertheless, it can still be noted that for one of the most abundant species *F. prausnitzii*, best hits obtained lower depth and template identity (Figure 1A; Supplementary material S11). For these exceptions, it is hypothesized that the reads from this species have split over the three *F. prausnitzii* templates available in the farmed_db, impairing the determination of a perfect match. This is supported by the data (Supplementary material S12) showing that the three *F. prausnitzii* templates [each assigned to a different clade (De Filippis et al., 2020) or species (Sakamoto et al., 2022)] from farmed_db were detected with a high depth but obtained various template identity results, with the reads covering only between 31 and 56% of the reference genomes. In comparison, when the GMS_db containing the exact GMS *F. prausnitzii* genome sequence (belonging to clade F) was used, a 100% template identity was obtained. For the remaining other best hits shown in Figure 1A, a decrease in depth (<4) was correlated to a decrease in template identity. This drop can be explained by the lower abundance of the species considered here and/or a matrix effect from the “spiked” sample.

A high depth (> 8) and template identity (> 90%) were reported for the detection of one of the GMS species, *E. coli*, in the “unspiked” sample sequenced by ONT singleplex (Experiment B) (Supplementary material S11). It is acknowledged that the presence of this species in the background of the chicken feces microbiota can bias the determination of TP results from the “spiked” GMS sample, resulting in possible overestimation. However, as *E. coli* is already present in high abundance (14%) in the GMS, this overestimation is considered to be limited.

The FP results for species detection were almost delimited by a maximum depth of 4 and a maximum template identity of 10% (Figure 1B). Surprisingly, one template identified as *Rhodococcus* sp. obtained a high percentage identity above 60% but with a depth below 2. This genus has often been reported as possible reagent contaminant in microbiome studies (Salter et al., 2014; Piro and Renard, 2023).

Considering the TP and FP data obtained for species detection with KMA, confidence thresholds were proposed to help with the interpretation of the data (Figure 1C). All the KMA results with depth and template identity equal or above 4 and 80%, respectively, were considered as positive, with nuance added to results with extremely good mapping scores, i.e., depth and template identity above 8 and 90%, respectively. Then, all the results with percentage identity below 10% were considered as likely negative. Intermediary thresholds were determined for all other results falling in a depth range of 0.1–4 and template identity range of 10–80%, following the trend of depth in relation with percentage identity for the TP values (Figure 1C).

Considering the results of Section 3.1, databases composed of complete genomes are preferred for KMA analysis. However, this was not possible in our study as no complete genomes belonging to the GMS species *P. corporis* and *V. rogosae* were available in RefSeq during analysis. Therefore, draft genomes were added to the farmed_db, which were able to detect these two bacterial species. To counterbalance the drawbacks of using draft genomes with KMA (shown in Section 3.1), a third criterion was included in the detection threshold: the template length. The average size of the complete bacterial genome templates from the farmed_db was approximately 10^6^ bp; if KMA reported a result from a template having a shorter length, this was penalized as a lack of genome coverage and identity and was considered as partial detection, with a lower level of confidence. Taking this into account, the final confidence thresholds and related results of interpretation guidelines for the detection of species with KMA were proposed (Table 3).

**Table 3:** Confidence threshold for the detection of bacterial species using KMA.

Confidence threshold			Level of confidence			Result interpretation
Depth	%ID	Length				
≥ 8	≥ 90	≥ 10^6^		6	High	The corresponding species is detected and identified with a high level of confidence.
4–8	80–90	10^5^–10^6^		5		
1–4	60–80	5 × 10^4^–10^5^		4	Medium	Partial detection, identification at species level to be confirmed.
0.5–1	40–60	10^4^–5 × 10^4^		3		
0.1–0.5	10–40	10^3^–10^4^		2	Low	Weak detection, result to be confirmed by alternative method.
0–0.1	0–10	0–10^3^		1		

ID%, Template identity in percent; length is expressed in bp.

Levels of confidence are numbered from 1 to 6, 6 being the most likely positive and 1 being the most likely negative, giving to the user various nuances in the interpretation of the results. Each level of confidence is determined by the combination of depth, template identity, and template length ranges, with first number included in the range and last number excluded. For instance, every hit reported by KMA with a depth higher or equal to 4 but lower than 8, a template identity higher or equal to 80% but lower than 90%, and a template length higher or equal to 10^5^ bp but lower than 10^6^ bp, obtained a level of confidence of 5, which informed the user that the species was detected with a high level of confidence.

The same methodology and samples were used for the determination of confidence thresholds for the detection of AMR genes by KMA with the ResFinder_db. There was a clear distinction between TP and FP, as shown in Figures 1D,E. Indeed, all the detected AMR genes, except for *aac(6′)-laa*, obtained a depth of above 4 and a template identity of above 97%. Some GMS AMR genes [*tet(Q)*, *tet(W)*, *mdf(A),* and *erm(B)*] were also detected with high mapping scores (depth > 4 and template identity >97%) in the “unspiked” sample, probably leading to a bias in the “spiked” GMS sample (Supplementary material S11). The *aac(6′)-laa* gene was detected with a lower depth comprised between 1.5 and 4 (Figure 1D), which was not surprising as this gene was carried by a GMS species present at 0.01% (*Salmonella enterica*) in the standard and could be detected only in the “pure” sample sequenced by ONT singleplex (see section 3.3). Very few FP results were reported (only in the ONT triplex condition), and they all obtained a depth of below 1.5 (Figure 1E). Based on these numbers, three levels of confidence threshold were proposed for the detection of AMR gene (Figure 1F), as shown in Table 4. As for the detection of species, an intermediary level between clear positive and negative detections was proposed, with a minimum depth of 1.5 and a minimum template identity of 90%, as this percentage identity value included all the TP and was frequently used in the literature for filtering KMA output data obtained with ResFinder (Brinch et al., 2020; Osakunor et al., 2020; Horie et al., 2021; Stege et al., 2022). As the variation in template length was less important among AMR genes than between complete and draft genomes, this criterion was not needed for the elaboration of confidence threshold for the identification of AMR genes.

**Table 4:** Confidence threshold for the detection of AMR gene using KMA.

Confidence threshold		Level of confidence			Result interpretation
Depth	%ID				
≥ 4	≥ 97		3	High	The corresponding AMR gene is detected with a high level of confidence.
1.5–4	90–97		2	Medium	Partial detection, allelic variant of the AMR gene to be confirmed.
0–1.5	0–90		1	Low	Weak detection, result to be confirmed by alternative method.

ID%: template identity in percent.

Each level of confidence is determined by the combination of a minimum depth and template identity. Levels of confidence are numbered from 1 to 3, 3 being the most likely positive and 1 being the most likely negative. For instance, every hit reported by KMA with a depth higher or equal to 1.5 but lower than 4 and a template identity higher or equal to 90% but lower than 97% obtained a level of confidence of 2, which informed the user that the species was detected with a medium level of confidence.

### 3.3 Evaluation of the feasibility of linking taxonomic classification and the detection of AMR genes with KMA

The feasibility of linking the taxonomic and AMR gene results obtained with KMA was investigated. A two-step KMA procedure was performed with the GMS “pure” long-read sequencing data obtained with ONT singleplex (Exp B). Table 5 shows the taxonomic identification and AMR genes that are detected using the GMS_db and ResFinder_db, respectively, as well as their association. Except for *S. enterica* and *E. faecalis*, a link between AMR genes and their host has been found for the other expected bacterial species, with high level of confidence. No linkage could be found for *E. faecalis* and its AMR gene *lsa(A)*, as the species and gene were not detected in the sample due to its low abundance (0.001%) in the GMS. Similarly for *S. enterica*, its AMR gene *aac(6′)-laa* was detected in the whole sample but could not be associated with its host as the species was not detected in the sequencing output. A few unexpected associations were found for *mdf(A)* and *tet(W),* but with a low level of confidence, in the range of detection where FP results are known to potentially occur. The two-step KMA procedure succeeded to retrieve the expected associations, although it was impacted by the relative abundance of the species in the GMS “pure” sample.

**Table 5:** Linking taxonomic and AMR gene detections in the GMS sample ‘pure’ sequenced with ONT singleplex (Exp B).

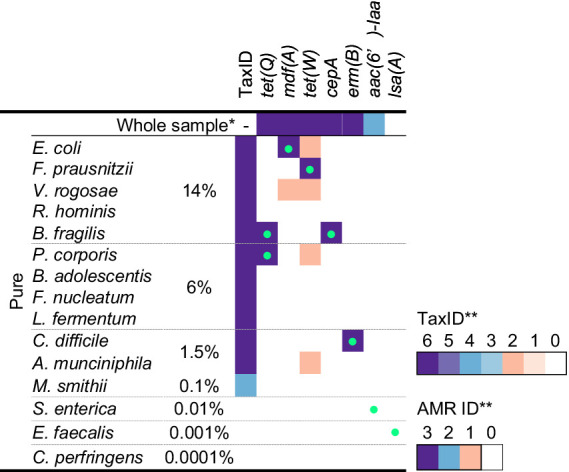

Table is showing at the left the KMA results obtained for taxonomic identification (TaxID) with GMS_db and at the top KMA results for AMR genes (*tet(Q), mdf(A), tet(W), cepA, erm(B), aac(6’)-laa, and lsa(A)*) identification (AMR ID) in the whole sample (*) with ResFinder_db. The list of GMS species is presented with their percentage of expected abundance. In the middle are shown with green dots (●) the expected associations between AMR genes and bacterial species carrying them, and the obtained AMR ID results, i.e., the AMR gene detection in reads from the corresponding bacterial species (2nd KMA step). ** Levels of confidence were high (6-5), medium (4-3) and low (1-2) for TaxID (see Table 3) and high (3), medium (2) and low (1) for AMR ID (see Table 4), no color (0) means no detection.

### 3.4 Validation of the KMA confidence threshold and AMR linkage strategy with different metagenomics datasets obtained from sequencing of mock communities with various experimental conditions

With the aim to further test the strategy elaborated in this study for KMA output data interpretation and AMR linking, several sequencing datasets, obtained with various experimental conditions including three mock communities, namely, GMS, MCSI, and MCSII, were analyzed with KMA using the farmed_db for the identification of bacterial taxonomic and the ResFinder_db for AMR for the detection of genes (Table 6; Supplementary material S13).

In general, the confidence levels reflected the correct detection of the GMS species and their AMR genes and the link with their host (Table 6). However, besides influenced by species relative abundance and/or the spiking into a matrix, as shown in Section 3.2, correct detection seemed to also be affected by experimental factors such as, the sequencing technology, the running time, and the level of multiplexing. Globally, the more samples were multiplexed (Experiments C, D, and E), the less data per sample were available (Supplementary material S4) and the more the confidence levels decreased for the detection of species and AMR genes, in comparison with singleplex experiments (Experiments B, F, and G). Notably, the detection of *S. enterica*, one of the lowest abundant GMS species (0.01%), linking with its AMR gene *aac(6′)-Iaa*, was obtained with medium level of confidence, with the “pure” sample processed with Experiments B and F, both in singleplex. The confidence levels generally decreased when the GMS was spiked into a matrix, illustrating the potential impact of the matrix on the quality of the DNA extraction (probably influenced by the DNA extraction kit used) and dilution of the GMS sequences in the background microbiome and matrix DNA. This was particularly clear for *B. adolescentis*, a gram-positive bacterium with high G/C content, which could not be clearly detected anymore in the “spiked” sample, independent of the tested conditions, whereas this species obtained good detection scores in the “pure” sample. As expected, *F. prausnitzii* never obtained a detection score higher than medium, despite its high relative abundance in the GMS (14%), which is in line with the mapping problem suspected for this species (section 3.2). Although the focus of this study was to develop confidence thresholds for long-read metagenomics data, the confidence levels were applied successfully to short-read sequencing data (Table 6). When comparing Illumina and ONT sequencing, both performed in singleplex (Experiments A and B), the two technologies were able to pick-up the same species from the GMS, except for *E. faecalis* which was only detected by Illumina at a low level of confidence. For the other detected species and the detection of AMR, comparable or higher level of confidence was generally obtained by ONT in comparison with Illumina. It can be noticed that no GMS species with an abundance 0.1% or lower could be detected with a confidence level of higher than 2 (low) in the “spiked” samples, suggesting that the limit of detection of the investigated metagenomics approaches was somewhere between 0.1 and 1.5% of genomic DNA in the sample, which corresponded to 4.79 × 10^6^ and 5.02 × 10^5^ cells, respectively (Supplementary material S1).

**Table 6:** Validation of the KMA interpretation and AMR linking strategy with GMS sequencing datasets obtained with various experimental settings.

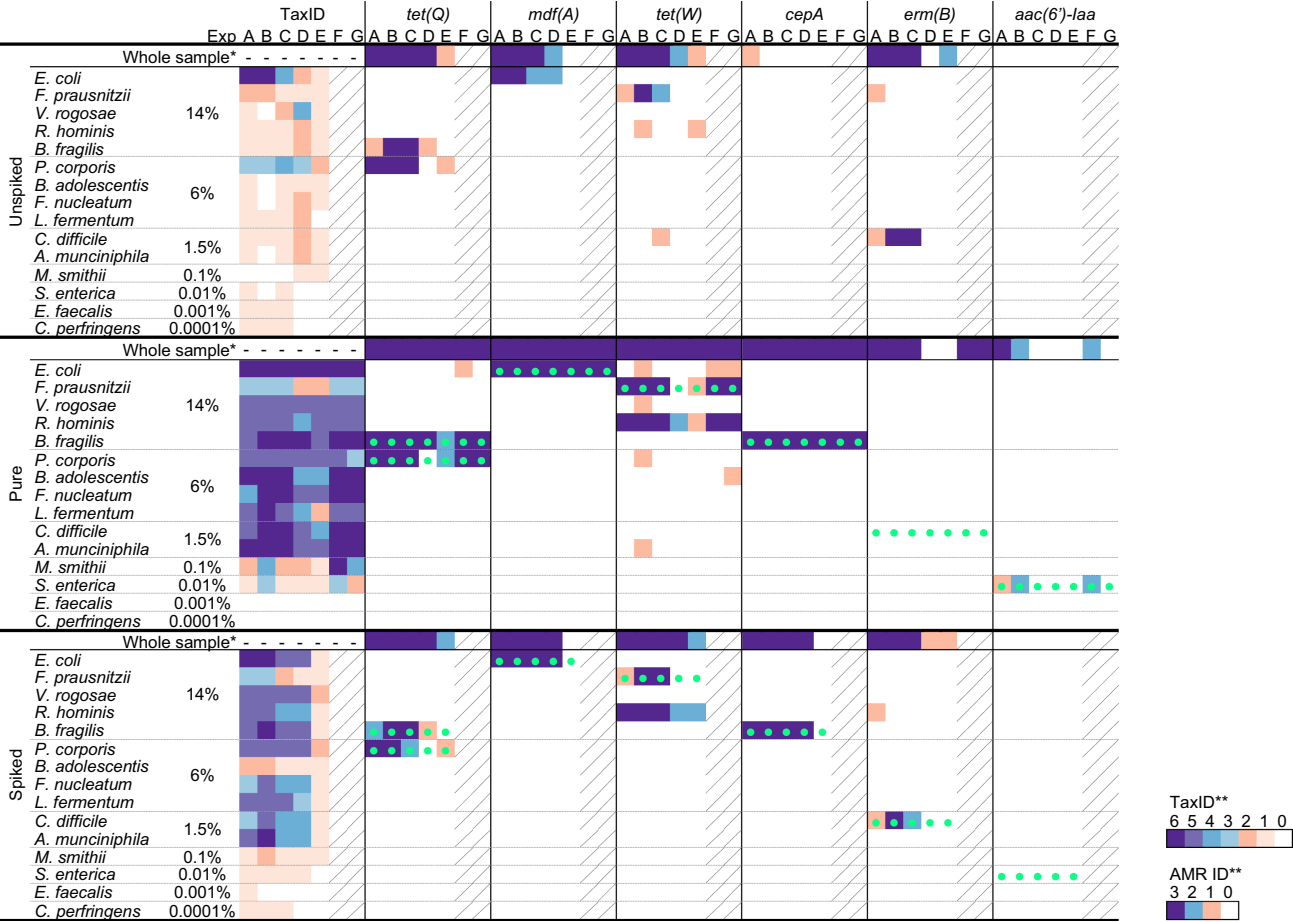

The table is showing at the left the KMA results obtained for taxonomic identification (TaxID) with farmed_db and at the top of ‘Unspiked’, ‘Pure’ and ‘Spiked’ sample rows, KMA results for AMR genes (*tet(Q), mdf(A), tet(W), cepA, erm(B) and aac(6’)-laa*) identification (AMR ID) in the whole sample (*) with ResFinder_db. The list of GMS species is presented with their percentage of expected abundance. In the middle are shown with green dots (●) the expected associations between AMR genes and bacterial species carrying them, and the obtained AMR ID results, i.e., the AMR genes detection in reads from the corresponding bacterial species (2nd KMA step). Experiments A to G include the GMS samples analyzed ‘pure’ or ‘spiked’ into a matrix, as well as the matrix processed alone ‘unspiked’ (see Table 1 for more details). For better readability, the lsa(A) results and potential association with E. faecalis were not shown as this AMR gene could never be detected in any of the tested conditions. ** Levels of confidence were high (6-5), medium (4-3) and low (1-2) for TaxID (see Table 3) and high (3), medium (2) and low (1) for AMR ID (see Table 4), no color (0) means no detection; /: not measured as in Exp F and G the GMS was analyzed pure only.

Two-step KMA analysis was successful in linking species and AMR genes, although it was affected by the experimental settings, with multiplexing likely decreasing the sensitivity of the method (Table 6). Surprisingly, no linking could be established between *erm(B)* and its host *C. difficile* in the “pure” sample, whereas this species and its AMR were correctly detected alone in most experimental conditions. On the contrary, this linking could be established in the “spiked” sample for Experiments A, B, and C because of the presence of erythromycin-resistant *C. difficile* in the background microflora, as the same AMR and species could be linked to the “unspiked” sample. Moreover, it can be noticed that independent of the experimental conditions, an unexpected link was established between *R. hominis* and *tet(W)* in the “pure” and “spiked” samples.

For the other mock community samples, i.e., MCSI (even) or MCSII (log), pure or spiked, extracted with different DNA purification methods (Table 1; Supplementary material S13), the attribution of the confidence levels for taxonomic identification and the detection of AMR genes matched the relative abundances of the two community standards. Indeed, only high levels were attributed for the MCSI (Experiment H; even distribution) and decreasing confidence levels were obtained for the MCSII (Experiments I, J, and K; log distribution), in line with the decreasing abundance of the species. Similarly, for the GMS experiments, the correct detection of the MCSII species was impacted by spiking into a matrix, the choice of DNA extraction method, and the amount of sequenced data (Experiments I, J, and K). With the MCSI and MCSII mock communities containing more AMR genes than the GMS, the two-step KMA could be further tested and proved again to be successful for the linking of AMR and identification of species, although this was negatively affected by the low abundance of *S. enterica*, *E. faecalis*, and *S. aureus* in the MCSII (Supplementary material S13). It can still be noticed that *fosX* and *crpP* never obtained a level of confidence higher than medium. This is due to the KMA results obtained for their template identity, being comprised between 90 and 97%. These results were in line with the percentage identity obtained with ResFinder when analyzing the MCS *S. enterica* and *L. fermentum* host reference genomes containing *fosX* and *crpP* (Supplementary material S7). The percentage identity not being above 97% for *fosX* and *crpP* detection, is probably due to their templates in the ResFinder database not being the same allelic variants than the ones from the *S. enterica* and *L. fermentum* strains contained in the MCSI and MCSII.

It can be noticed that some FP results were reported for the identification of species and AMR gene when analyzing the “pure” GMS sample over the seven experimental conditions (Experiments A to G) and the pure MCSI and MCSII samples with Experiments H and I (Supplementary material S14). In almost all cases, a low level of confidence was attributed to the KMA output data for these results. In few cases for the detection of AMR with Experiments D and E, some FP obtained the detection of medium and high levels of confidence. Experiments D and E involved high level of multiplexing, where “pure,” “spiked,” and “unspiked” samples were barcoded and mixed together. Therefore, it is suspected that FP with such high mapping scores, i.e., depth between 2 and 13 and template identity between 95 and 100%, come from crosstalk of the barcoding, as shown in other studies (Xu et al., 2018; Hatfield et al., 2020).

Finally, the dataset of Exp F was chosen to evaluate the performance of the two-step KMA procedure with farmed_db and ResFinder_db when used on a virtual laptop, simulating a more “on-site” environment. This dataset was chosen because it contained the highest number of bases (Supplementary material S4) and included the highest number of expected species (14 bacteria and one archaeon) from the GMS, for which potential linkage with AMR genes could be investigated, being the most challenging. The first KMA analysis for the detection of species with the farmed_db took 59 min 12 s to be completed and did not require more than 6.06 GB of RAM. The subsequent operation of extraction of the reads, belonging to one of the GMS species, followed by second KMA analysis with the ResFinder_db and extracted reads as input, took 54 min to be completed with an average of 3 min 36 s per species. The total two-step KMA procedure took 1 h 52 min to be completed for Experiment F.

### 3.5 Application of the KMA confidence threshold on publicly available metagenomics datasets in the context of food safety

*k*-mer alignment analysis, including AMR linking, was performed with the farmed_db and ResFinder_db on four metagenomic datasets generated by ONT sequencing of food enzyme products (Coobra, Stillspirits, Browin, and Pureferm) containing three GMM *Bacillus* species, i.e., *B. licheniformis*, *B. amyloliquefaciens,* and *B. velezensis* (D’aes et al., 2022) and their related AMR genes, i.e., *clbA, blaP, erm(D),* and *aadD* [referred to as *ant(4′)-Ia* in the original study], *bleO*, and *catA*. The KMA level of confidence developed in this study was applied to the KMA output data to determine if the targeted species and their related AMR genes could be detected in the case of original studies, where other data analysis tools had been used (Buytaers et al., 2021; D’aes et al., 2022). The detection statistics reported in the original study are shown in Supplementary material S15 compared with the obtained KMA interpretation results. As expected, the attributed KMA levels of confidence decreased in line with the depth values obtained in the original study when mapping sequencing reads to *Bacillus* reference genomes (Supplementary material S15). Moreover, high level of confidence was attributed to species for which viable isolates or Metagenome-Assembled Genomes (MAGs) with level of completeness of above 70% were obtained in the original study. On the contrary, when no viable isolates or MAGs with high completeness could be found, lower level of detection was attributed to the concerned species (Supplementary material S15).

The *erm(D)* gene was reported being common in *B. (para)licheniformis* strains (Agersø et al., 2018; Zhou et al., 2019; D’aes et al., 2022). This was illustrated here by its detection and linkage to *B. licheniformis* with high level of confidence in all samples except one (Pureferm), in which it was also not detected by D’aes et al. (Supplementary material S15). The *aadD* gene was correctly detected in all the samples but could not be linked to its two bacterial hosts, except for *B. velezensis* in the Pureferm sample. This can be explained by the fact that this AMR gene was used as a reporter in the genetic artificial constructs, which were suggested to be located on a plasmid originating from *Staphylococcus aureus* (D’aes et al., 2022). As obtained in the original study, the *aph(3′)-Ia* gene was detected in the PureFerm sample with high level of confidence but could not be linked to one of the *Bacillus species* (data not shown). Finally, few other bacterial species and AMR genes were detected as FP, with a low level of confidence (Supplementary material S16).

The KMA analysis was also performed with the farmed_db on long-read metagenomics sequencing data from minced beef sample containing a pathogenic *E. coli* (STEC) strain (Buytaers et al., 2021). The sequencing data were generated with a MinION or Flongle device. When performing Flongle sequencing, a protocol for the removal of matrix (host) DNA was also tested. For all three conditions, *E. coli* could successfully be detected with high level of confidence (Supplementary material S17). Additionally, the template hit that obtained the best mapping score came from the genome (NC_017906.1) of an O157:H7 STEC strain isolated from a Chinese outbreak (Xiong et al., 2012), which was corresponded to the expected serotype and pathotype of the *E. coli* strain present in the minced beef sample. Moreover, the KMA interpretation thresholds helped to visualize and compare the different experimental conditions. As expected, Flongle sequencing, with 10 times less output, was less powerful than MinION but could be improved for some species by the use of a host DNA removal technology (Supplementary material S17). The same background genus, as detected in the original study, could also be retrieved here. The presence of potential false positive could not be evaluated as the exact composition of the minced beef microbiome was not fully known. As no AMR was previously reported for the STEC strain, the presence of AMR genes was not investigated here.

## 4 Discussion

Shotgun metagenomics is a promising method that revolutionized the world of pathogen diagnostics and surveillance with an untargeted approach for the detection of microorganisms and their AMR profiles (Hendriksen et al., 2019; Kirstahler et al., 2021). Combined with the ease of use of ONT long-read real-time sequencing, it has the potential to improve the rapid detection of pathogens along the food chain and their AMR genes and how they spread in food-producing environments. To allow this, performant bioinformatics tools, such as KMA, with tailored databases, are needed to process the huge amount of generated data and deliver the interpretation of clear and simple results. In this study, we elaborated a filtering strategy based on detection thresholds with confidence levels and proposed guidelines, aiming to help future KMA users to obtain a clear interpretation of their metagenomics data. The suggested thresholds were validated with 28 metagenomics datasets, which were obtained from the sequencing of real or spiked samples. The composition of those samples was known, either via the use of defined microbial mock communities or because the samples had been previously analyzed using other methods (e.g., taxonomic classification with Kraken2 or metagenomic hybrid assembly followed by whole genome alignment-based comparison). This allowed the verification of the defined confidence thresholds. Moreover, a two-step KMA-based approach was explored to link AMR genes and bacterial species, and this strategy was validated with the same sequencing datasets.

The reference genomic database used with taxonomic classifiers is a key aspect of the bioinformatics analysis performed on metagenomics data. In our study, we showed that the use of a database containing draft genomes, made of several contigs with various nucleotide lengths, was not compatible for simple and straightforward interpretation of the KMA output. Indeed, the filtering strategy developed in the present study was based on the attribution of a confidence level of the detection of a single template sequence, which was linked to a bacterial genome. Therefore, this was not adapted to draft genomes fragmented in several template sequences, which would need to be compiled together for proper interpretation. Furthermore, short contigs were more likely to obtain high depth and percentage identity, which could have been misleading for the users, hampering the interpretation of correct results. Previous studies already suggested that the incompleteness of genomes in databases decreases the reliability and accuracy of metagenomics by making bioinformatics tools report false positive results which require to be filtered out (Lu and Salzberg, 2018; Wright et al., 2023). Although the increasing number of genome submission on public repositories contributes to the sequencing effort to enrich the databases with more and more organisms, draft genomes are sometimes highly fragmented and can contain artifacts if not properly curated (Breitwieser et al., 2019). However, using reference complete genomes only is not always possible for some organisms; no complete circular genomes are available yet. Most of the time, complete genomes are available only for a limited number of well-studied bacterial species (Smith et al., 2022). Although this should not impair pathogen detection, as complete genomes are usually available, this can impact microbiome studies in the context of AMR transmission between less sequenced and characterized commensal bacteria in food-producing environments. Therefore, to allow the use of incomplete genomes in a KMA database, if unavoidable, and avoid additional contig compilation/concatenation steps, which might be less straightforward for users with less bioinformatics skills, the template length was included in the criteria, which was used to determine the confidence thresholds elaborated in this study. For future KMA developments, it would be interesting to improve the performance of this tool when dealing with draft genomes, to avoid the bias introduced by the analysis of short sequences from fragmented genomes. Concerning the confidence threshold for the detection of AMR, it was not needed to include the template length in the criteria, as the genes are considerably shorter and complete sequences are always available.

Choosing the “optimal” database for taxonomic classification of metagenomics sequencing data remains a challenge. The ideal database is composed of as many organisms as possible, and not limited to bacteria, but also including animals, fungi, viruses, plants, and plasmids. However, the size of such a database would require enormous computational resources to be used with bioinformatics tools and would make analysis impossible or too long to complete. Moreover, a highly resource demanding analysis would not be compatible with the portability of ONT sequencing, with data analysis most likely being performed on a laptop if carried out on-site. On the other hand, it has been proven that small databases only composed of organisms of interest, e.g., of specific pathogens, and can lead to erroneous results such as false positives and negatives (Marcelino et al., 2020; Wright et al., 2023). This was also suggested in the present study, given the unexpected linkage obtained for *R. hominis* and *tet(W)*. This discrepancy can be attributed to the existence of only one reference template from this species in the farmed_db, which does not share the same AMR gene content as the strain present in the GMS. This finding aligns with observations by Bloemen et al. (2023). The lack of sufficiently similar genomic sequences as the template in the database can also lead to incorrect mapping, as illustrated by the insufficient coverage obtained for *F. prausnitzii*. This was due to the spread of reads over three different templates, which belong to different clades, without a perfect match. In addition, one template sequence (CP022479.1) has been recently re-identified as *Faecalibacterium duncaniae* by its submitter on NCBI. A good balance has to be found between a database as diverse as possible, containing good representation of genomes from the organisms most likely present in the analyzed sample (including DNA from the matrix/host if not removed beforehand), and a database that can be handled by non-expert users on laptops and that is also compatible with computationally efficient pipelines for fast processing of ONT sequencing data.

In addition to using appropriate databases to avoid erroneous results, another challenge of taxonomic classifiers, such as KMA, is the exclusion of false positive results while keeping a good sensitivity (Ye et al., 2019). This can be achieved by the configuration of particular options during the analysis, such as the minimum template identity (-ID) in KMA. Alternatively, we made the choice in this study to keep this parameter loose, with post-processing filtering of the KMA output data in mind, to identify the best hits per species and AMR allelic variant. However, as shown in the present study and in the literature, false positive results can be reported by read classifiers in the same depth and template identity ranges when detected species or AMR genes are present in low abundance in metagenomics samples (Ye et al., 2019; Xu et al., 2023). Therefore, filtering out these false positives by enforcing strict settings might also result in loss of sensitivity as some true positives could be discarded (Sun et al., 2021; Portik et al., 2022). This was experienced by Rooney et al. (2022) who tested different filtering strategies on KMA output data to improve AMR gene detection, resulting in a decrease in false positives, and the detriment of true positives. To avoid this, instead of setting a minimum threshold that will eliminate part of the results, confidence detection levels were elaborated in the present study, allowing an increase in the specificity of KMA without sacrificing its sensitivity. As such, each detection threshold is associated with a level of confidence, aiming to help the user with a clear and simple result interpretation. For instance, if detection with a low level of confidence is reported, the recommendation for complementary analyses that could confirm or reject the detection, such as real-time PCR, is provided to the user. Furthermore, in the case of partial genome/AMR gene coverage or if read mapping issues occur, the partial identification is communicated to the user with an intermediary level of confidence (medium). This was observed in this study for *F. prausnitzii,* with the spread of reads over several templates and also shown by Rooney et al. with the reads mapping to multiple AMR allelic variants (Rooney et al., 2022). To keep the interpretation of the KMA results straightforward, the more stringent template identity criterion was preferred over the template coverage for the determination of confidence threshold. Trustful identification is guaranteed by high template identity, which implies high template coverage when using KMA. When a low template identity is obtained, which will result in a medium or low level of confidence, investigation of the detailed KMA results is needed to check whether this is due to a lack of template coverage or query identity. The user can also try other complementary analyses, such as mapping of the reads against a reference genome belonging to the species or AMR gene that was not detected with high level of confidence, to confirm or reject partial identification with KMA.

Although the KMA confidence thresholds selected in our study were determined with only two samples processed each in two sequencing conditions, they were successfully tested on 28 sequencing datasets. Two types of datasets were used, but each time the content was known, either by using defined microbial mock communities or previously determined using other methods. Hence, a comparison could be made with the results obtained applying the thresholds, thereby validating those thresholds. Similar findings were found for the identification of taxonomic and AMR gene, in the context of GMM and pathogen detection for food safety. This confirmed that they can be applied to such types of metagenomics studies. Based on the confidence thresholds developed in this study, the CCMetagen tool could be extended to bacteria as only thresholds for fungi were proposed (Marcelino et al., 2020). For the characterization of pathogens, a similar approach, as was carried out for the AMR genes, could be performed for the detection of virulence genes with a dedicated database that could be developed and validated for the investigation of virulence factors. When the KMA-based thresholds were tested on other publicly available datasets including mock communities, they offered an alternative to the often used read/base count estimation, thereby facilitating the interpretation by giving a certain confidence level to the detection. Furthermore, although it was not the goal to analyze in detail the influence of the different experimental settings on the correct detection of the mock community species, the confidence threshold developed in this study helped to compare samples which were processed with different DNA extraction and sequencing protocols. Indeed, they demonstrated that the correct detection of the species contained in the mock communities was clearly impacted by some experimental settings, such as level of multiplexing (and hence barcoding and amount of generated data per sample), running time, spiking into a matrix, and/or basecalling model (fast accuracy vs. high accuracy). The confidence thresholds were successfully applied to short-read data. Nevertheless, this was tested with only one Illumina dataset, and further investigation is needed to verify if confidence thresholds optimized for ONT are fully compatible with Illumina sequencing data. While the thresholds determining the confidence levels were selected to be compatible with various experimental designs, they might need to be adapted to consider variations in metagenomics protocols. For instance, as the depth value is directly impacted by the level of multiplexing, the depth thresholds could be lowered to be adapted to high multiplexing conditions. Nonetheless, the risk would be to increase the occurrence of potential false positive results without detecting the low-abundant true positives. It is acknowledged that the “best detection thresholds” do not exist and that some adaptions might be needed, according to specific types of microorganisms studied, sample matrices, reference databases, and research questions (Wright et al., 2023). The different factors mentioned above, impacting the sensitivity of the metagenomics method, should be considered for future development of cost-efficient and time-efficient detection of AMR genes and their bacterial hosts, which are obtained in real-time and on-site by ONT sequencing. Fortunately, the ONT technology is rapidly evolving and constantly improving, with the new v14 chemistry and R10 flow cells, both allowing to achieve an accuracy above Q20, which will increase the detection performance of the method.

In addition to the KMA interpretation guidelines proposed in this study, a two-step KMA-based approach was also explored to link AMR gene detection and bacterial taxonomic identification. This included the detection of AMR genes present in reads, which were previously classified at the taxonomic level. It was successfully tested on two mock communities, containing seven and 12 AMR genes, respectively, under different experimental conditions. Furthermore, when simulating its usage on a laptop with a large dataset, the full procedure could be completed in less than 2 h, proving its potential for on-site application without remote access to powerful servers. The two-step KMA-based approach was also tested on sequencing datasets from the food enzyme products containing GMM *Bacillus* species and AMR genes but showed its limits when it came to the linking of an AMR gene that was suggested to be located in a plasmid. Although this workflow for AMR linking has to be further tested including more datasets with known content, it has the potential to add a new feature to the surveillance of AMR genes that are chromosomally located. Concerning AMR genes located on a plasmid, they cannot be easily linked to a host species without additional experimental steps, such as Hi-C sequencing (Brito, 2021; Lloréns-Rico et al., 2022). Finally, although the two-step KMA procedure was mainly developed and tested for long-read sequence data, it was also successfully applied to one short-read sequencing dataset. Nevertheless, more tests are needed to validate the method with short-read sequences.

Long-read sequencing, combined with clear and easy interpretation of the data due to confidence thresholds, allowed the detection of bacteria and their chromosomal AMR genes in complex metagenomics samples. We strongly believe that our approach has the potential to improve the surveillance of pathogens and AMR genes and the understanding of how they spread in natural microbiomes from commensal bacteria to pathogens. This is in line with priorities determined by the EU who stated in a recent report that AMR is a silent pandemic that remains a serious challenge (OECD, 2022). To assess the variability which can be encountered in different One-health applications, future studies will focus on further testing the approach on various real samples collected in food-producing environment, such as farms where portable ONT sequencing could be used.

## Data availability statement

All data generated or analyzed during this study are included in this published article and its supplementary information files. The dataset supporting the conclusions of this article is available in the NCBI Sequence Read Archive (SRA) repository, under the BioProject ID PRJNA1031997. The KMA result files (.res) as well as the KMA databases are available upon request.

## Ethics statement

Ethical approval was not required for the study, involving animals in accordance with the local legislation and institutional requirements because only animal fecal material was used and collected from environment.

## Author contributions

MG: Conceptualization, Data curation, Formal analysis, Investigation, Methodology, Resources, Validation, Visualization, Writing – original draft. IN: Investigation, Resources, Writing – review & editing. L-JB: Investigation, Resources, Writing – review & editing. JG: Data curation, Funding acquisition, Resources, Supervision, Writing – review & editing. SO-P: Data curation, Funding acquisition, Resources, Writing – review & editing. AR: Data curation, Resources, Writing – review & editing. SO: Data curation, Funding acquisition, Resources, Software, Writing – review & editing. VM: Funding acquisition, Resources, Writing – review & editing. BM: Resources, Writing – review & editing. BG-Z: Funding acquisition, Writing – review & editing. MB: Funding acquisition, Resources, Writing – review & editing. LM: Funding acquisition, Resources, Writing – review & editing. BB: Resources, Writing – review & editing. KV: Resources, Software, Writing – review & editing. NR: Funding acquisition, Resources, Writing – review & editing. MA: Funding acquisition, Project administration, Resources, Supervision, Writing – review & editing. SDK: Conceptualization, Funding acquisition, Methodology, Project administration, Resources, Supervision, Validation, Writing – original draft.

## Footnotes

^1^ftp://ftp.ncbi.nlm.nih.gov/genomes/genbank
