# Supplementary material for: Towards facilitated interpretation of shotgun metagenomics long-read sequencing data analyzed with KMA for the detection of bacterial pathogens and their antimicrobial resistance genes
Source: Front Microbiol. 2024 Apr 4;15:1336532. doi: 10.3389/fmicb.2024.1336532 (PMC11042533; doi:10.3389/fmicb.2024.1336532)
Supplement: Supplementary file 1 [file Data_Sheet_1.zip › Supplementary File 8.docx]

**Supplementary file S8: Details of the experimental settings A, D and E used for the generation of the GMS sequencing data**

For all experiments, the GMS was analyzed alone (pure) or spiked into a matrix (spiked). The matrix alone (unspiked) was also analyzed. All the filtered sequencing data were deposited in the NCBI SRA repository, under the BioProject ID PRJNA1031997.

1. Experiment A

The same ‘pure’, ‘spiked’ and ‘unspiked’ samples were processed for DNA extraction as for Exp B and C (see section 2.2 in the manuscript). Then, the library preparation was performed using the Nextera XT kit (Illumina, San Diego, USA) and sequenced on a MiSeq instrument (Illumina, Sand Diego, USA), generating paired-end reads of 250 bp with the reagent kit v3. The samples (‘pure’, ‘spiked’ and ‘unspiked’) were processed in singleplex with one sample per MiSeq run. The raw sequencing reads were filtered with Trimmomatic version 0.36 (Bolger, Lohse, and Usadel 2014) using the following parameters: ILLUMINACLIP:NexteraPE-PE.fa:2:20:10, LEADING:10, TRAILING:10, SLIDINGWINDOW:4:20, MINLEN:30.

1. Experiment D

For ‘spiked’ (matrix_mock) and ‘unspiked’ (matrix_blank) samples 75 µl of the GMS (≈ 2.96×108 cells in total) or PBS were inoculated into 100 mg of Buffalo feces material, respectively and mixed thoroughly before DNA extraction. For the ‘pure’ sample (mock_blank) 75µl of the GMS were mixed with 100 µl PBS. Total genomic DNA was extracted using Quick-DNA HMW MagBead kit (Zymo Research, Irvine, USA) as described in section 2.2 in the manuscript. The resulting DNA was measured using Invitrogen Qubit 4 Fluorometer (Thermo Fisher Scientific, Waltham, USA) with Qubit dsDNA HS Assay Kit (Thermo Fisher Scientific, Waltham, USA) and the Fragment Analyzer (Agilent Technologies, Santa Clara, USA). The resulting DNA quantity and average fragment size was 21 ng/µl and 18 000 bp for ‘pure’, 46 ng/µl and 20 000 bp for ‘spiked’ and 22 ng/µl and 14 000 bp for ‘unspiked’. The library preparation was performed using the Ligation sequencing kit for genomic DNA (SQK-LSK109) (Oxford Nanopore Technologies, Oxford, UK) with the native barcoding expansion kit (EXP-NBD104) and long fragment buffer (LFB) for multiplexing of 5 samples and 1 µg of DNA as total input. The resulting library was loaded on a MinION flowcell (version R9.4.1) and sequenced with the Mk1C device (Oxford Nanopore Technologies, Oxford, UK) for 48 hours. After basecalling and demultiplexing the reads were filtered with nanofilt (v2.8.0) for a minimum quality of 7 and a min read length of 1000 bp and trimmed with porechop (v0.2.4) with a min score of 60.

1. Experiment E

The samples originated from a UK pig farm study conducted in 2017 [1], and stored at -80^o^C. The ‘spiked’ sample was prepared by mixing 75µl of GMS with 200µl DNA/RNA shield (2x) and 200mg pig fecal material followed by throughout homogenization. The ‘pure’ sample was only 75ul GMS and the ‘unspiked’ sample was 200mg pig fecal material.

The ‘pure’, ‘spiked’ and ‘unspiked’ samples were processed using GenFind v3 kit (Beckman Coulter) with the protocol modified to enable extractions from fecal samples. These modifications included: i) 200mg of fecal sample used for lysis; ii) MetaPolyzyme (Sigma-Aldrich) was used instead of the lysozyme; iii) RNase A was not used in the lysis step; iv) at the binding stage, following the addition of Bind (BBB) solution, the sample was placed on a Hula mixer and incubated at room temperature for 30min. A total of six samples were multiplexed using the Rapid barcoding kit (SQK-RBK004) and sequenced on MinION Mk1B using the R9.4 flow cell (Oxford Nanopore Technologies). High accuracy basecalling was enabled using Guppy 5.0.17 with the option to trim sequencing adapters enabled and the quality threshold set to 9. Following sequencing, no additional data filtering or trimming was performed.

1. AbuOun M, Jones H, Stubberfield E, Gilson D, Shaw LP, Hubbard ATM, et al. A genomic epidemiological study shows that prevalence of antimicrobial resistance in Enterobacterales is associated with the livestock host, as well as antimicrobial usage. Microb Genom. 2021;7(10); doi: 10.1099/mgen.0.000630.
