# Supplementary material for: Towards facilitated interpretation of shotgun metagenomics long-read sequencing data analyzed with KMA for the detection of bacterial pathogens and their antimicrobial resistance genes
Source: Front Microbiol. 2024 Apr 4;15:1336532. doi: 10.3389/fmicb.2024.1336532 (PMC11042533; doi:10.3389/fmicb.2024.1336532)
Supplement: Supplementary file 1 [file Data_Sheet_1.zip › Supplementary File 9.docx]

**Supplementary file S9: Depth and template identity are influenced by the template length and the composition of the database**

**
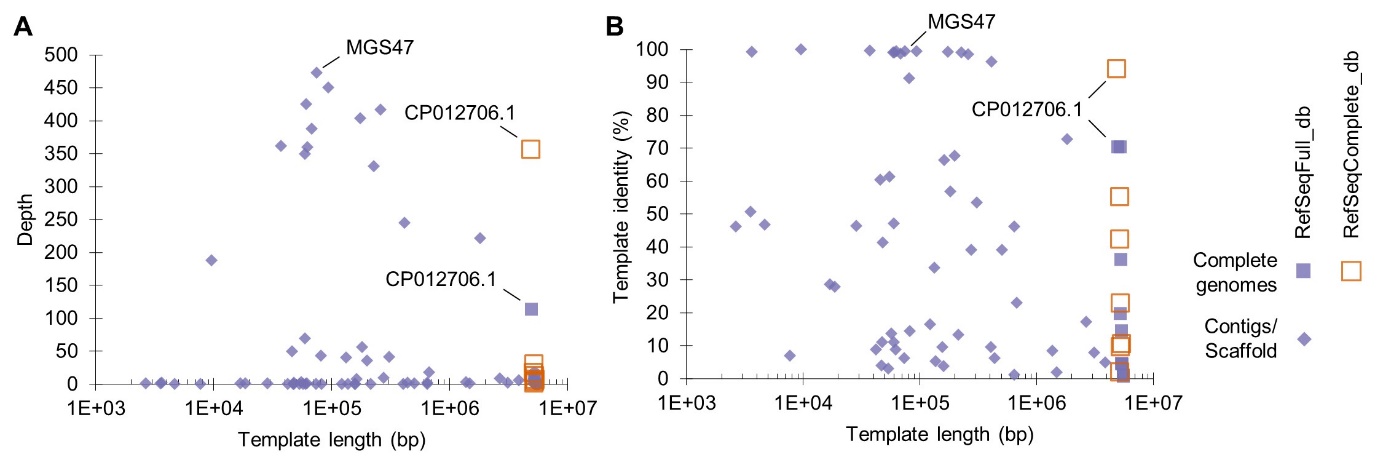
**

Sequencing data obtained from ‘pure’ sample processed with ONT singleplex were analyzed by KMA using RefSeqFull_db and RefSeqComplete_db. For each hits generated by KMA, the graphs show the representation of the depth (y-axis in A) and template identity (y-axis in B) in function of the template length (x-axis in log scale) from the reads that have mapped against *Bacteroides fragilis* template sequences. CP012706.1 and MGS47 (scaffold 84) represent the best hit template names from the *B. fragilis* template sequences available in the two tested databases. A database with only complete genomes (RefSeqComplete_db) allows a better interpretation of the result with a clear perfect match, while with a database made of draft genomes (RefSeqFull_db ), the reads are spread among contigs and scaffold, hampering result interpretation.
